# Cemented Calcar-Guided Short-Stem Prostheses in Geriatric Patients: Short-Term Results from a Prospective Observational Study

**DOI:** 10.3390/antibiotics13080739

**Published:** 2024-08-06

**Authors:** Bertram Regenbrecht, Ahmed Yaseen, Gideon Wagener, Michael Wild

**Affiliations:** 1 Klinik Lilienthal GmbH & Co. KG, 28865 Lilienthal, Germany; 2 Universitätsklinik für Orthopädie und Traumatologie, A-6020 Innsbruck, Austria; 3 Department of Orthopedics, Trauma and Hand Surgery, Klinikum Darmstadt, 64283 Darmstadt, Germany

**Keywords:** total hip arthroplasty, antibiotic-loaded bone cement, cemented short stem, clinical study

## Abstract

Both cementless and cemented stems have exhibited favorable long-term outcomes in total hip arthroplasty. Nonetheless, in elderly patients, cemented hips offer an advantage due to their reduced risk of periprosthetic fractures. This study aimed to assess the initial outcomes of 28 patients who underwent unilateral cemented total hip arthroplasty utilizing a calcar-guided A2 stem (ARTIQO GmbH, Lüdinghausen, Germany). Various types of antibiotic-loaded bone cement were employed. During follow-up, we recorded demographic data and comorbidities and employed standardized clinical assessment tools, including the Harris Hip Score. Radiographic assessments included preoperative, postoperative, and follow-up imaging to evaluate subsidence, osteolysis, and bone resorption. The results indicated that among the 28 patients, 5 withdrew consent and 2 patients passed away from unrelated causes. Additionally, one prosthesis was explanted due to the undersizing of the cement stopper, which resulted in an inadequate cement mantle. As a result, 20 patients underwent a 1-year follow-up, revealing noteworthy enhancements in clinical scores, with no instances of radiolucent lines or osteolysis. No infections were detected. In summary, our short-term experience with this particular cemented short-stem design yielded promising results, exhibiting excellent functional outcomes, no aseptic loosening attributable to the stem, and no infections. Further clinical studies and registry data are essential to corroborate these findings.

## 1. Introduction

Total hip arthroplasty is one of the most successful surgical interventions, generally reporting excellent outcomes and low complication rates [1]. The evolution of modern cemented joint arthroplasty traces back to Charnley, who initiated the use of acrylic bone cement for securing femoral head prostheses within the femur in 1958 [2]. Polymethyl methacrylate (PMMA) is a synthetic polymer commonly used in medical applications. PMMA bone cement possesses unique mechanical properties that make it an ideal material for the fixation of implants in orthopedic surgery [3]. These properties include biomechanical stability, as PMMA cement quickly stabilizes the implant in bone tissue, providing immediate fixation and anchorage. It also improves load distribution on the contact surface between the implant and bone, reducing stress concentrations. PMMA cement fills and levels off the implant–bone interface, enhancing the fit and stability of the implant within the bone. Additionally, it stiffens the spongiosa around the implant, further improving the anchorage of the implant in the bone. PMMA cement is easy to apply during surgery, allowing for the efficient and effective fixation of implants. Furthermore, PMMA has been proven to be nontoxic and compatible with human tissue, ensuring biocompatibility and reducing the risk of adverse reactions [3].

In 1970, Buchholz and Engelbrecht were the first to explore the potential of incorporating antibiotics into bone cement [4]. The use of antibiotic-loaded bone cement (ALBC) as a local antibiotic delivery system in orthopedic surgery has yielded significant findings. ALBC achieves high local antibiotic concentrations at the implant–cement–bone interface, ensuring targeted effects and minimizing systemic burden, thus reducing adverse effects. It is effective in preventing prosthetic joint infections due to its broad-spectrum antimicrobial effect. Antibiotics are released following diffusion, sustaining their activity. Combination therapies, such as gentamicin with clindamycin, further enhance antimicrobial efficacy. PMMA bone cement serves as an effective local antibiotic delivery system that is crucial in preventing infections in orthopedic surgery [3]. 

Although cemented hip arthroplasty remains largely standard in the treatment of medial femoral neck fractures in older individuals in traumatology [5], the introduction of cementless arthroplasty, particularly with proximally anchoring straight stem prostheses, has experienced a significant surge [6,7]. The literature has shown that cementless and cemented stems exhibit good long-term outcomes in hip arthroplasty, with comparable survival rates [8,9]. However, the limits of cementless arthroplasty are evident, particularly in older patients, as shown by increased complications, especially periprosthetic fractures and infections. The German Hip Registry (EPRD) shows a divergence of 1% in terms of excess cumulative revision probability with follow-up times of up to 7 years [10].

To mitigate the elevated risk of periprosthetic fractures, the exploration of alternative prosthetic options is warranted. In this context, one measure recommended by the German EPRD advocates the utilization of cemented hip stems in elective procedures for patients over 75 years old [10]. This recommendation is supported by the literature, demonstrating a reduced risk associated with cemented stems compared to cementless hips in this age population [11,12,13]. Research further suggests that the choice of stem design significantly influences periprosthetic fracture occurrence in total hip arthroplasty (THA), with short stems generally posing a lower risk than straight stems [12,14]. Although the adoption of cementless short stems has rapidly expanded in recent years [15], cemented short stems remain comparatively less established within the orthopedic community. Given their limited availability and utilization, their conceptualization and adoption within the orthopedic field are notably nascent.

In our clinic, a tertiary care endoprosthesis center with approximately 500 hip-arthroplasty procedures performed last year, the proportion of cemented hip arthroplasty procedures has steadily increased in recent years, reaching approximately 20%. This shift occurred after a period during which cementless short-stem endoprostheses were almost exclusively used in elective cases, while cemented standard stems were consistently used in trauma cases.

Since 2021, a calcar-guided cemented short-stem model (A2 stem, ARTIQO, Lüdinghausen, Germany) has been available in our clinic and utilized in both elective and trauma cases (the latter in combination with a bipolar head). Approximately 160 cemented short-stem prostheses have been implanted in our clinic since their introduction. Of these, 28 patients were recruited to participate in a larger prospective multicenter study, which included a total of 121 patients overall. This prospective study was designed to collect clinical and radiographic data on the A2 stem after up to 10 years of follow-up.

An interim analysis of internal clinical data, including EPRD manufacturer data, revealed that although the overall revision rates for primary hip arthroplasty are low, cemented standard stems exhibit slightly higher revision rates. This may be attributed, at least in part, to patient selection criteria favoring older patients and those with a poorer bone quality. Interestingly, cemented short stems do not contribute to the revision rates associated with cemented standard stems in elective and trauma cases, suggesting potentially lower complication rates than expected.

The aim of the current study was to assess both the clinical outcomes and complications associated with the use of this cemented short-stem design.

## 2. Materials and Methods

This prospective study adhered to the principles of the Helsinki Declaration and obtained approval from the ethics committee of the Medical Association of Lower Saxony, Germany. The study did not restrict inclusion to specific indications within the intended use of the prosthesis. From September 2021 to April 2023, a total of 28 unilateral cemented THA procedures were conducted using an A2 stem and a cementless ANA.NOVA Alpha Cup (Implantec, Mödling, Austria). Different antibiotic-loaded cement types were used during this patient series, including Palacos^®^ R + G (Heraeus Medical, Wehrheim, Germany) and Optipac^®^ (ZimmerBiomet, Warsaw, IN, USA). The cohort consisted of 25 females and 3 males, with an average age of 80.6 ± 4.3 (range, 71.0–90.0) years and an average weight of 68.2 ± 10.5 (range, 48.0–90.0) kg. The indications for implantation were primary osteoarthritis (*n* = 27) and rheumatoid arthritis (*n* = 1).

**Figure 1:**
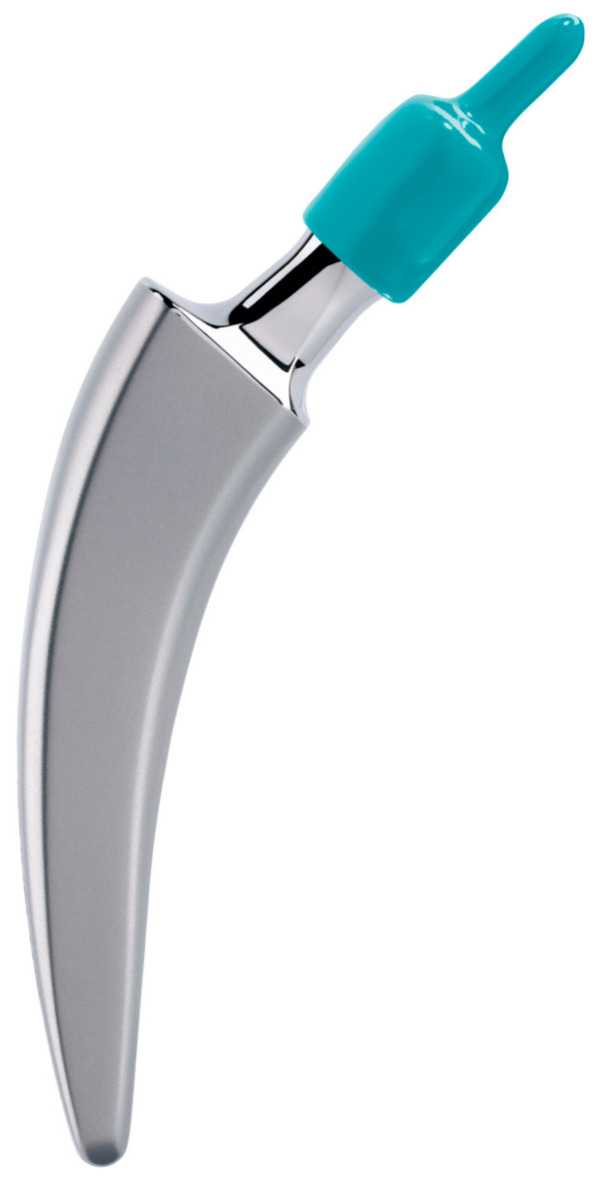
The cemented A2 stem (courtesy of ARTIQO).

The CE-marked cemented calcar-guided A2 stem (Figure 1) was developed based on the design philosophy of the cementless A2 short stem. It is constructed from a stainless-steel implant compliant with the ISO 5832-9 standard and features a smooth surface that allows for the use of bone cement. This stem is intended for use in trauma patients with displaced femoral neck fractures or in older individuals. Moreover, the cemented stem serves as an intraoperative backup solution in cases where the primary stability of the cementless version may be inadequate, such as instances of poor bone quality. The stem is available in nine sizes, ranging from 2 to 10. The instrument set is universally applicable and remains identical for both the cemented and cementless versions.

All surgeries utilized a minimally invasive direct anterior approach [16]. The stem can be implanted using either an “undersizing” cementing technique (where the stem is one size smaller than the rasp) or a “line-to-line” technique (where the stem and rasp are identical in size). In our clinic, all stems were implanted using the undersizing technique. Following cup implantation, the femoral canal was prepared, and trial reduction ensured an optimal range of motion, joint stability, and correct leg length. The cementation procedure was performed according to a contemporary technique described in the literature [17]. In brief, a CEMSTOP^®^ cement restrictor (Teknimed, Vic-en-Bigorre, France) was introduced into the femoral medullary canal. The cement restrictor was positioned 1 cm distal to the tip of the prosthesis. To prepare the bone bed, the femoral medullary canal was flushed with pulse lavage. The high-viscosity bone cement (PALACOS^®^, Heraeus Medical GmbH, Wehrheim, Germany) was mixed at room temperature using a vacuum system for approximately 30 s. Subsequently, at the beginning of minute 2, the cement was retrogradely introduced into the femoral canal using a cement gun with a pressure holder. The cement curing was observed for 12 min.

Following surgery, the patients were encouraged to bear weight fully on the operated leg from the first postoperative day, with physiotherapy initiated thereafter.

The demographic data, comorbidities, Harris Hip Score (HHS) [18], and patient-administered HOOS-Physical Function Shortform (HOOS-PS) [19] were assessed preoperatively and during follow-up, whereas the Forgotten Joint Score (FJS) [20] was used during follow-up. Patient activity was determined using the UCLA Activity Scale (UCLA) [21]. Radiographic evaluation included standing anterior–posterior and lateral radiographs of the proximal femur preoperatively, immediately postoperatively, and during follow-up, analyzing subsidence, osteolysis, and bone resorption using Gruen zones [22]. Periarticular ossification was classified according to the Brooker classification [23]. Leg-length inequality was assessed radiographically and clinically. The rehabilitation protocol included immediate weight bearing.

### Statistics

For continuous variables, the data were presented as mean ± standard deviation (SD); for categorical variables, the data were presented as counts.

## 3. Results

Of the 28 patients who underwent surgery, 5 withdrew their consent and 2 patients died due to unrelated causes. One prosthesis was explanted at 2 months postoperatively due to undersizing of the cement stopper, resulting in an inadequate cement mantle, which subsequently led to 14 mm of subsidence in the weeks following implantation. Hence, there were 20 patients with a 1-year follow-up (Table 1).

**Figure 2:**
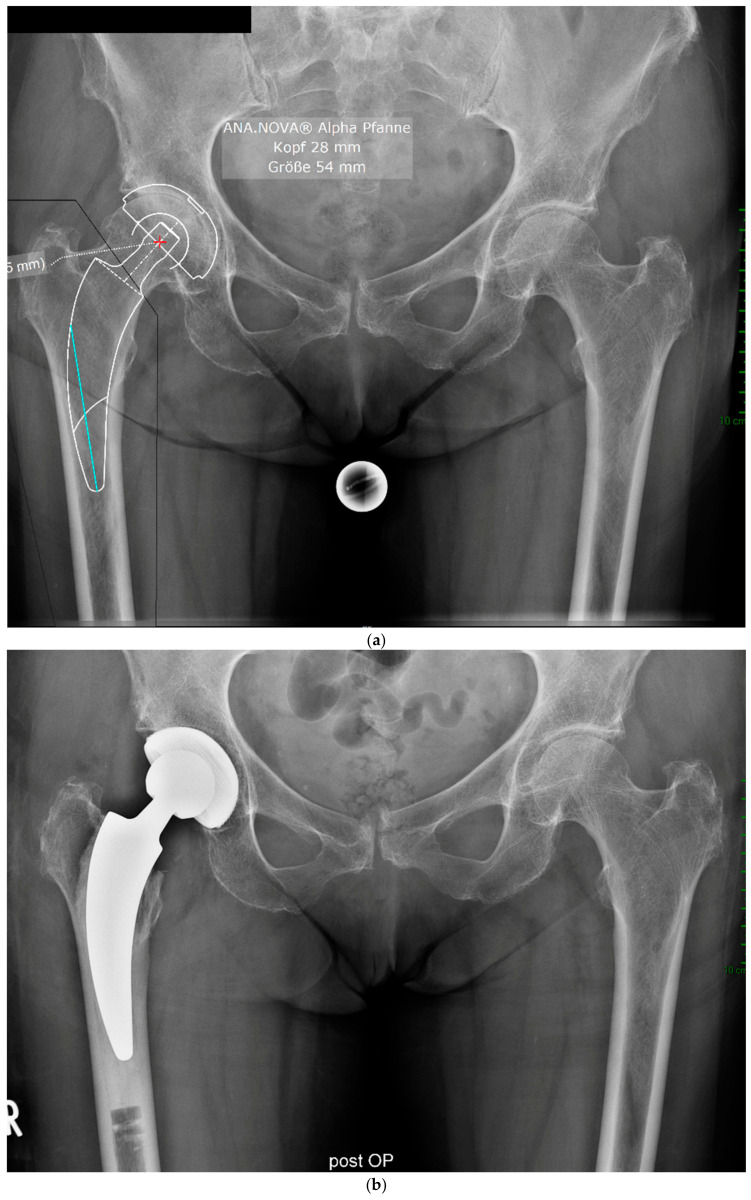

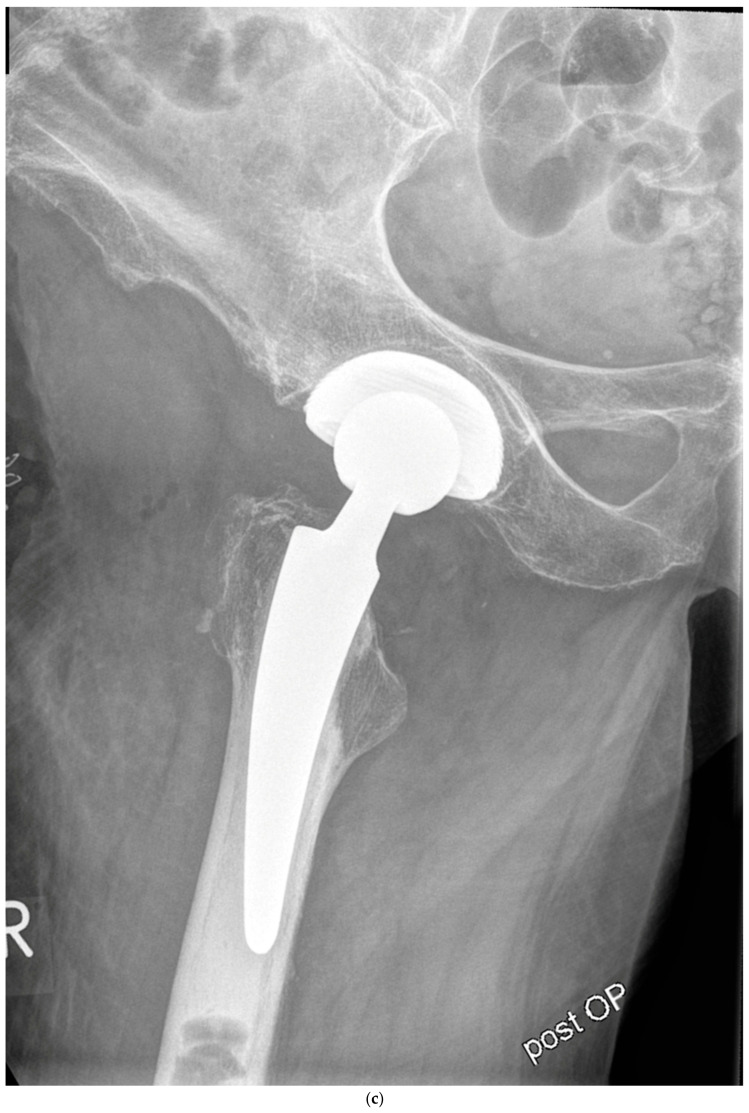
Case #1: Female patient, 81 years old, with osteoporotic bone, coxa vara, and a Dorr C femur. (**a**) Preoperative planning for a cemented A2 stem combined with a cementless ANA.NOVA Alpha Cup. The image shows good reconstruction of the leg length and offset. (**b**,**c**) Postoperative anteroposterior (**b**) and axial (**c**) radiographic imaging 1 week postoperatively. Correct position of the stem and complete cement mantle.

**Figure 3:**
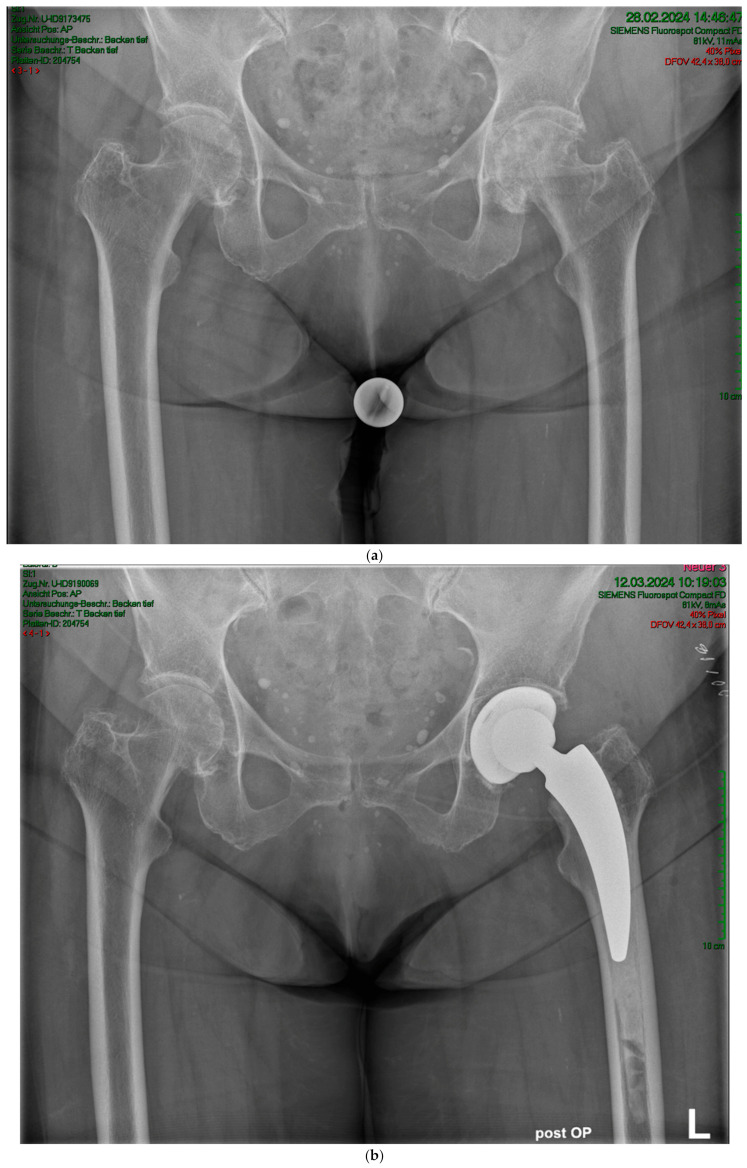

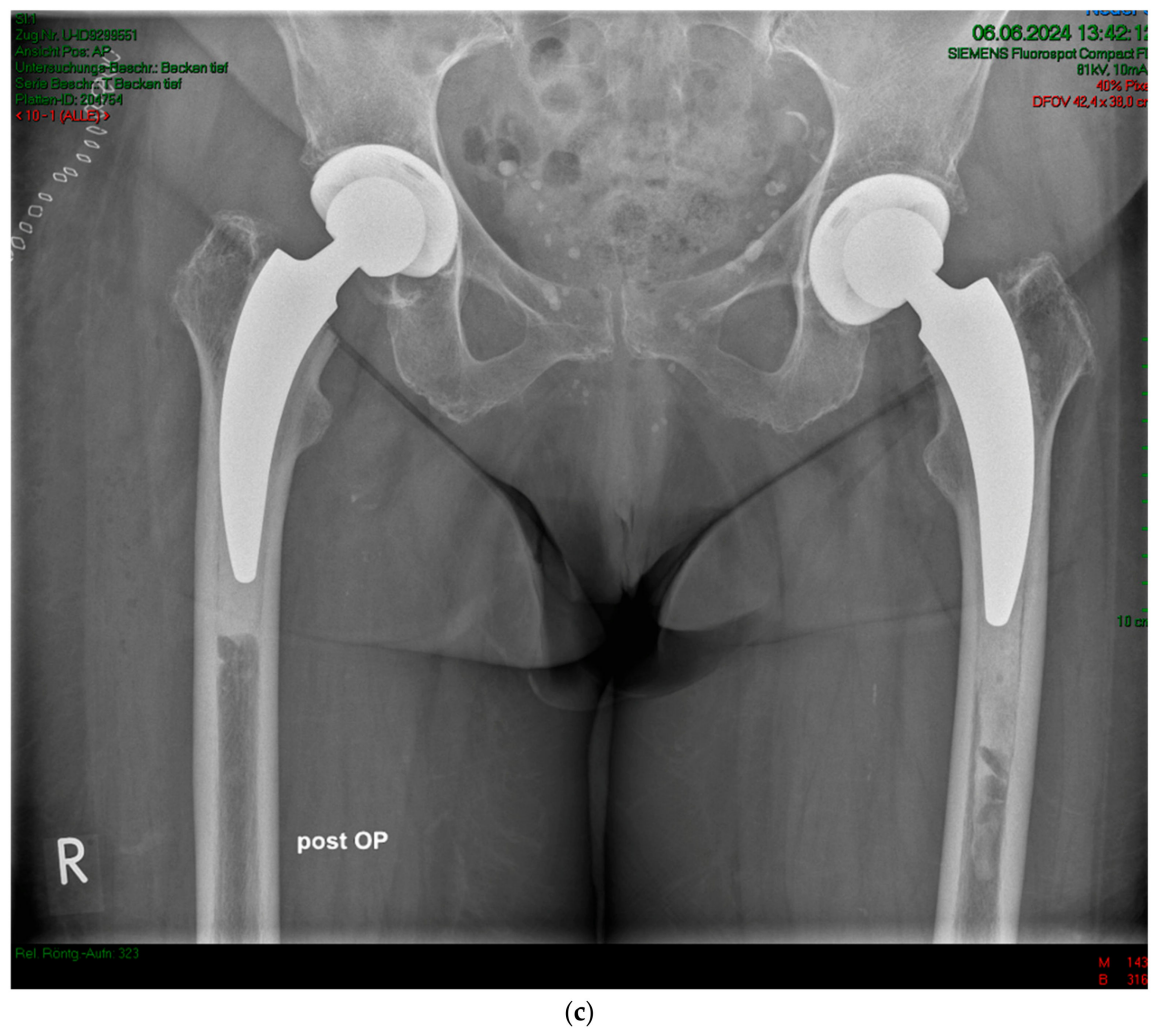
(**a**) Case #2: Osteoarthritis, female, 85 years old, bilateral sequential implantation of an A2 stem, Dorr B-C femora. (**b**) Left side: slight distalization of the cement stopper. (**c**) Right side: no positional change of the cement stopper during the operation.

**Figure 4:**
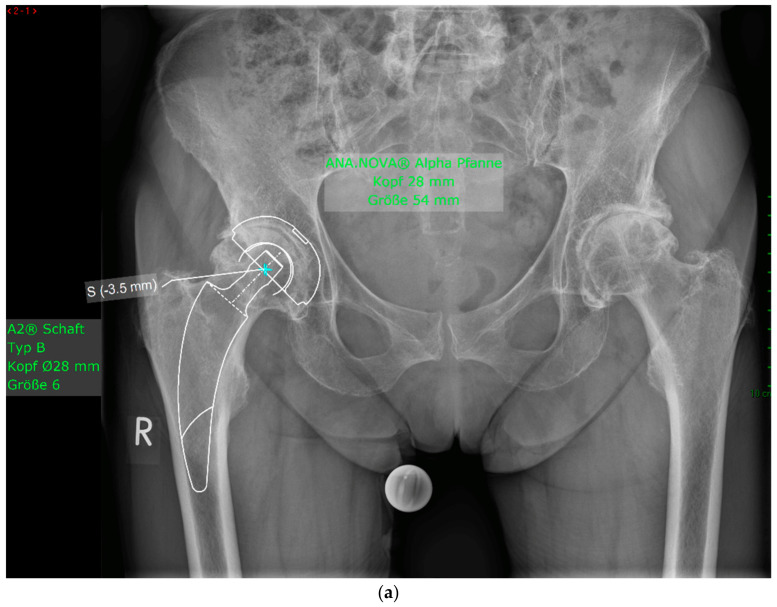

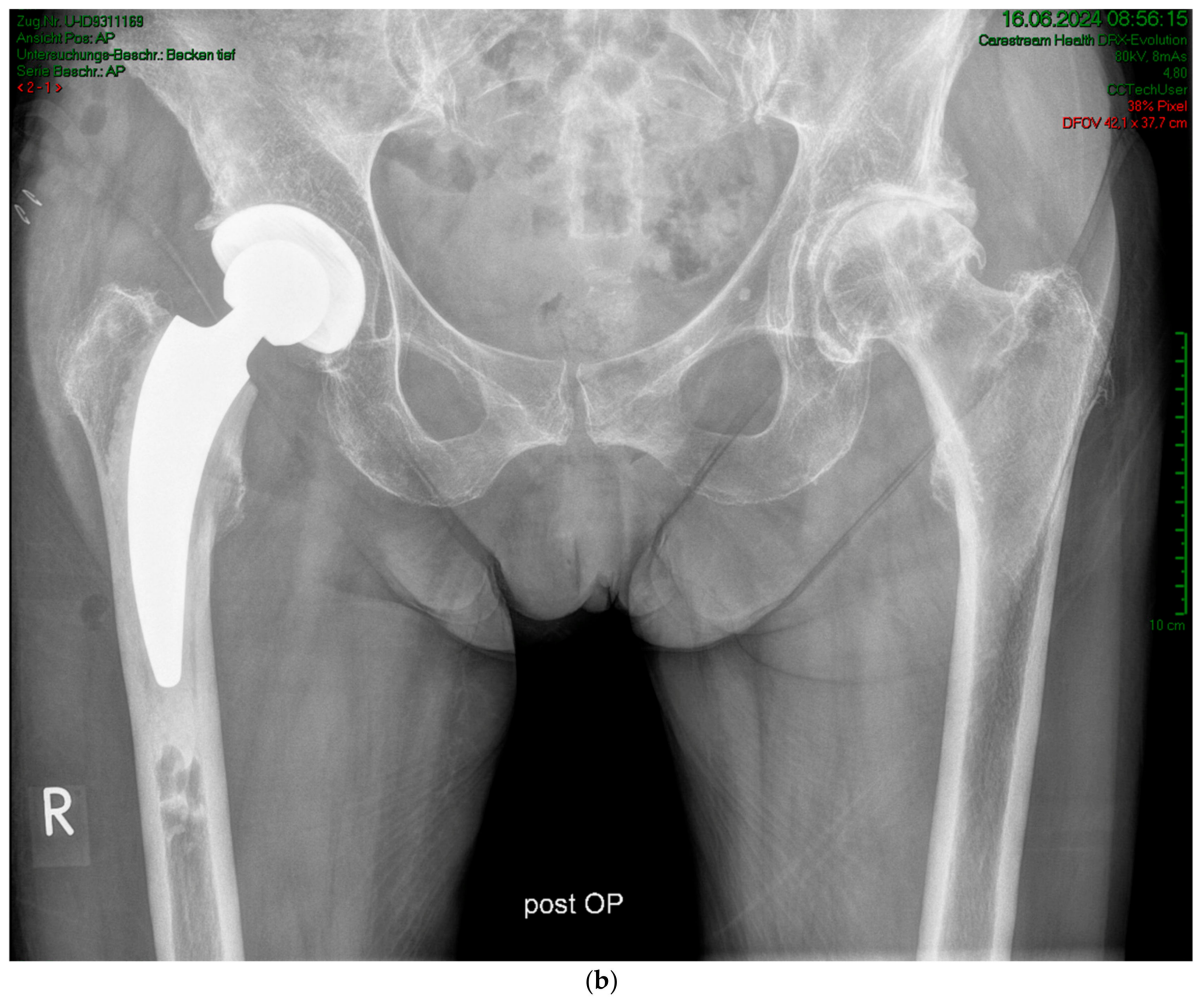
(**a**) Case #3: Osteoarthritis, female, 81 years old, preoperative planning. (**b**) Correct implant position 1 week postoperatively. Homogeneous cement mantle.

The mean HHS increased from 49.4 ± 13.9 preoperatively to 93.6 ± 6.9 at the final follow-up. No pain was reported by 19 patients at the final follow-up, with only occasional slight pain in one patient. The mean UCLA score increased from 3.5 ± 1.5 preoperatively to 5.1 ± 1.9 at the one-year follow-up. The mean HOOS-PS score increased from 44.2 ± 22.1 preoperatively to 60.0 ± 28.6 at the one-year follow-up. The FJS score was 68.3 ± 14.6 at the one-year follow-up. One patient experienced leg shortening of 10 mm. Periarticular ossification Brooker 1 was identified in three patients. One patient experienced axial subsidence of 2 mm. There were no postoperative infections. No radiolucent lines were observed, and none of the patients presented with osteolysis. A representative case is presented. Preoperative planning facilitated the achievement of anatomic restoration, as depicted in Figure 2. Figure 3 and Figure 4 demonstrate the correct postoperative positioning of the femoral stem and a complete cement mantle.

## 4. Discussion

Calcar-guided short-stem total hip arthroplasty is rapidly advancing in hip reconstruction, combining the benefits of short-stem implants with a minimally invasive surgical technique. Cementless calcar-guided stems have been shown to offer stem stability, preserve bone stock, and optimize the anatomical reconstruction of offset, antetorsion, and the antetilt of the femoral neck [15,24,25].

Although various studies have reported good outcomes using the uncemented version of the A2 short-stem femoral implant [15,25], this series represents the first reported utilization of the cemented femoral variant.

We observed favorable short-term results in elderly patients undergoing primary THA using a short calcar-guided cemented stem. The clinical scores, including the HHS, UCLA, and HOOS-PS, showed significant improvements postoperatively. These improvements are consistent with the existing literature, where both short-stem and conventional stems typically show increased HHS after surgery. However, a meta-analysis found no significant differences in HHS between short-stem and conventional stem prostheses [26].

Furthermore, a recent study by Van Beers et al. reported similar HHS improvements at one year but observed better HOOS values in their cohort using uncemented prostheses, which had a mean age of 60 years compared to the mean age of 81.3 years in our study [27]. We attribute the slightly better HOOS outcomes in Van Beers et al.’s study to the younger cohort.

Given the increasingly aging population in Western industrialized nations, coupled with the high demand of this elderly population for maintaining or regaining mobility into advanced age, as well as age-related high morbidity and reduced immune competence, the question of whether cemented THA offers any advantages in geriatric patients arises. In the elderly, the risk of periprosthetic femoral fracture with cementless total hip arthroplasty, especially in Dorr C femora [28], increases due to deteriorating bone quality [29]. Moreover, the risk of infection increases with advancing age and multimorbidity. Significantly reduced infection rates by approximately 30% with cementless short-stem prostheses compared with standard stem prostheses have been reported [15]. By using a cemented short-stem prosthesis, an additional layer of protection against infection may be added. In hip arthroplasty, the use of ALBC has been shown to contribute significantly to infection reduction [30]. When implants are introduced, there is a risk of biofilm formation on the implant surface, which can lead to chronic infections [31]. Systemic antibiotics may not effectively prevent colonization of the implant. However, locally released antibiotics can provide high concentrations at the implant site, protecting the foreign material from microbial contamination. PMMA bone cement is commonly used as a carrier for local antibiotic therapy. A meta-analysis conducted in the United States demonstrated that the application of ALBC can reduce infection rates by approximately 50% in primary hip arthroplasty [30]. Individual patient considerations should guide treatment decisions, but the use of ALBC remains an essential measure to mitigate infections associated with implants [31].

From a biomechanical perspective, employing a short cemented stem is feasible, although it requires a flawless bone cement interface attainable through modern cementation methods. In their biomechanical study on osteoporotic bone, Azari et al. demonstrated that the line-to-line technique and the undersized technique are equivalent and that both techniques are suitable for calcar-guided short stems [32]. The authors concluded that regardless of the specific cementation technique, cemented short stems appear promising in patients with low bone quality [32].

Cemented calcar-guided short-stem THA offers conceptual advantages over standard cemented THA. By virtue of their reduced dimensions compared to standard THA, short-stem designs necessitate a reduced insertion force. This enables the introduction of bone cement at a stage characterized by higher viscosity, thereby guaranteeing the secure fixation of the implant within the medullary cavity. In comparison to standard THA, in cemented short-stem THA, the cement volume is lower and the bony surface that comes in contact with the cement involves cancellous metaphyseal bone rather than dense diaphyseal cortical bone. These differences result in lower intraosseous pressures due to the enhanced permeability of the cement into the metaphyseal spongy bone. In our own biomechanical study, we observed that the use of an A2-cemented short stem compared with a cemented straight stem resulted in a reduction in intramedullary pressure [33]. Although high intramedullary pressure per se is an important factor in the genesis of bone cement implantation syndrome, the extent of embolization is proportional to the intramedullary pressure [34].

In our study, we focused on evaluating the short-term outcomes of cemented calcar-guided short-stem THA specifically within our cohort. The limitations of the current study include its relatively small sample size and lack of statistical power to adequately detect and assess perioperative complications. Additionally, the follow-up period was relatively short, potentially limiting the ability to assess long-term outcomes and complications associated with the cemented short-stem design.

Although we reported improvements in clinical scores such as HHS, UCLA, and HOOS, it is important to note that these scores are general measures of functional status and are influenced by a range of factors beyond the type of prosthesis used. Comparing these scores with those from other studies is challenging due to differences in baseline patient characteristics, surgical settings, and follow-up protocols, making it difficult to isolate the impact of the prosthesis from other factors confounding clinical outcomes. While a control group would have been appropriate to directly compare outcomes with alternative treatment modalities or standard practices, we did not include one, thereby limiting the interpretability of the results in the context of broader clinical practice.

As a result, while our findings provide preliminary insights, further research with larger cohorts and extended follow-up is necessary to more comprehensively evaluate the effectiveness and safety of the cemented short-stem prosthesis.

In summary, our short-term experience with this particular cemented short stem with ALBC is promising, demonstrating excellent functional outcomes, no infections, and no aseptic loosening attributable to the stem design. This series highlights the critical role of modern cementing techniques, including jet lavage and cement pressurization, alongside proper cement-stopper utilization in achieving successful outcomes in THA. Leveraging preliminary data from ongoing in vitro studies suggesting reduced periprosthetic complications associated with ALBC fixation in short-stem THA, these implant designs warrant further investigation as a potential solution for the elderly population undergoing THA. However, further studies, including larger cohorts and registry data, are needed to confirm these assertions and determine their clinical relevance.

## Figures and Tables

**Table 1:** Baseline characteristics of patients evaluated at 1-year follow-up.

Variable	Value
Gender (female/male) *	19/1
Age [years] (mean ± std. dev [range])	81.3 ± 4.5 (71–90)
Weight [kg] (mean ± std. dev [range])	67.6 ± 11.0 (48–90)
BMI [kg/m^2^] (mean ± std. dev [range])	24.3 ± 3.2 (19.2–31.1)
Indication for implantation: * - Primary osteoarthritis - Rheumatoid arthritis	27 1

* Presented as number of observations. Abbreviation: std. dev.: standard deviation.

## Data Availability

The data are not publicly available for privacy reasons.
